# Leukotriene B4 as a Potential Therapeutic Target for the Treatment of Metabolic Disorders

**DOI:** 10.3389/fimmu.2015.00515

**Published:** 2015-10-08

**Authors:** Luciano Ribeiro Filgueiras, C. Henrique Serezani, Sonia Jancar

**Affiliations:** ^1^Institute of Biomedical Science, University of São Paulo, São Paulo, Brazil; ^2^Department of Microbiology and Immunology, Indiana University School of Medicine, Indianapolis, IN, USA

**Keywords:** Leukotriene B4, Diabetes, metabolic disorders, sterile inflammation, insulin resistance

In the last decade, the incidence of metabolic disorders has increased drastically worldwide and is becoming a global health threat. Studies have shown that the pathogenesis and co-morbidities of diseases such as diabetes, gout, and atherosclerosis involve chronic low-grade inflammation and metabolic changes (1). As this inflammation is triggered by endogenous substances, instead of pathogens, it is called “sterile inflammation”. Chronic low-grade inflammation can be triggered by the accumulation of metabolic products such as uric acid, glucose, cholesterol, and free circulating fatty acids. These substances can induce inflammation by two distinct mechanisms: (1) engagement of Toll-Like Receptors (TLR), such as TLR-2 (2), TLR-4 (3), and TLR-9 (4) and (2) activation of the intracellular receptor complex known as inflammasome that leads to caspase-1 activation, an enzyme that cleaves pro- interleukin (IL)-1β into its active form (5–7). IL-1β acts on its receptor IL1R1, a member of the TLR family whose activation is dependent on the presence of the adaptor molecule Myeloid Differentiation primary response gene 88 (MyD88). Although TLR-2 signaling is mediated mainly through the MyD88, TLR-4 activates MyD88-dependent and TIR-domain-containing adapter-inducing interferon β (TRIF)-dependent pathways. The MyD88-dependent pathway culminates in the activation of the Nuclear Factor kappa B (NFκB)/Activator Protein (AP) 1 and the TRIF-dependent pathway leads to delayed activation of NFκB associated with Interferon Regulatory Factor (IRF) (8). Thus, NFκB is a transcription factor of several genes involved in inflammation and also regulates its own transcription (9). In metabolic diseases with chronic low-grade inflammation, NFκB is continuously activated (10). Since NFκB can be activated through the adaptor molecule MyD88, modulation of its expression should have important consequences on the inflammatory response.

Leukotrienes are lipid mediators whose production is increased during inflammation. Activated phospholipase A2 releases arachidonic acid from membrane phospholipids. Liberated (soluble) arachidonic acid can be metabolized by 5-lipoxygenase (5-LO) to produce leukotrienes including LTB_4_ and cysteinyl leukotrienes, LTC4, LTD4, and LTE4. It is well documented that leukotrienes are mediators of inflammatory events such as edema and leukocyte infiltration and activation and that they have an essential role in acute and chronic inflammatory diseases. Leukotrienes were also shown to mediate resistance to infections by several microorganisms (11). In macrophages, leukotrienes were shown to potentiate phagocytosis and microbicidal activity by affecting the mechanisms involved in actin polymerization and activation of NADPH oxidase, respectively (12).

LTB_4_ binds to two distinct G protein-coupled receptors. The Leukotriene Receptor (BLT)1 is the high affinity receptor that induces inflammation, enhances cytokine production, phagocytosis, and mediates antimicrobial effector functions. Through BLT1, LTB_4_ was shown to enhance MyD88 expression and potentiate MyD88-dependent stimuli responses while no difference on MyD88-independent stimuli was found (13). BLT2 binds LTB_4_ with lower affinity and has been much less studied, currently no information is available on BLT2 in the context with metabolic syndrome. It was shown that LTB_4_ through both, BLT2 and BLT1 receptors enhances NFκB activation (14).

It can be concluded that LTB_4_, by increasing MyD88 expression, would potentiate a TLR/IL-1R dependent sterile inflammation. Considering that metabolic diseases involve sterile inflammation we propose that LTB_4_ plays a central role in the development of metabolic diseases and may be considered a target for the development of new therapies. Here, we will highlight recent findings on LTB_4_ involvement in Type 1 Diabetes (T1D), Type 2 Diabetes (T2D), and gout.

According to the World Health Organization, diabetes is a syndrome characterized by hyperglycemia with disturbances in protein, lipid, and carbohydrate metabolism due to a deficiency in insulin production (in T1D) or insulin resistance (in T2D). In T1D, both hyperglycemia and insulin deficiency can be responsible for the sterile inflammation (15, 16). We found that mice with T1D exhibited higher serum levels of IL-1β, TNF-α, and LTB_4_. Macrophages from type 1 diabetic mice, compared to those from non-diabetics, expressed higher levels of MyD88 mRNA and produced higher levels of pro-inflammatory cytokines and nitric oxide, in response to MyD88-dependent stimuli such as LPS and IL-1β. Inhibition of LT synthesis restored MyD88 expression and cytokines production to similar levels found in macrophages from non-diabetic mice (15). Another important finding in this work was that pharmacologic or genetic inhibition of LTB4/BLT1 protected mice from succumbing to sepsis and this correlated with decreased macrophage MyD88 expression and decreased systemic inflammatory responses in the septic mice. This was an interesting finding because increased susceptibility to sepsis is a characteristic of diabetic patients (17).

In T2D, obesity is one of the largest risk factors for the development of insulin resistance (18, 19). It has been proposed that in obese people and in murine models of obesity, chronic sterile inflammation is triggered by free fatty acids (FFA), which engage MyD88-dependent receptors to produce IL-6 (20) and TNF-α (21). FFA can also activate the inflammasome and induce IL-1β production (7). Macrophages that infiltrate adipose tissue seem to play an essential role in insulin resistance. In diet-induced obesity, adipose tissue macrophages express an activated M1 phenotype (22–24). These results suggest that pro-inflammatory cytokines produced by macrophages have a local effect on adipocytes and a systemic effect on liver and muscle cells impairing insulin signaling.

In obese mice, increased uptake of omega-3-polyunsaturated fatty acids (ω-3-PUFA) led to enhanced insulin sensitivity. This correlated with decreased production of 5-LO products and increased generation of anti-inflammatory lipid mediators such as resolvins and protectins in the adipose tissue (25). Resolvins and protectins are mediators derived from ω-3-PUFA and are associated with the resolution phase of inflammation (26). Resolvin E1 can bind to BLT1, acting as a partial agonist to attenuate LTB_4_-induced NFκB activation in polymorphonuclear leukocytes. The effect of resolvin E1 was comparable to that of the BLT1 antagonist, U-75302 (27). Together these results suggest a dominant role for LTB_4_ through BLT1 in insulin resistance.

Recently, it was demonstrated that knockdown of the Ltb4r1 gene (the gene that transcribes BLT1) or inhibition of LTB_4_ synthesis protected mice from diet-induced insulin resistance (10, 28, 29). In mice fed a high-fat diet, increased amounts of LTB_4_ can be found in the white adipose tissue, liver, and muscle (29, 30). In obese animals, LTB_4_ promotes NFκB p65 nuclear translocation and production of IL-6 and TNF-α in adipose tissue (10). Moreover, when NFκB activation is increased, LTB_4_ could enhance pro-IL-1β expression for subsequent cleavage to the mature form via inflammasome activation.

Another possibility is that in skeletal muscle cells, adipocytes, and hepatocytes, LTB_4_ by enhancing MyD88 expression and action would potentiate the IL-1R response, further impairing insulin signaling in insulin target organs. LTB_4_ was also shown to decrease insulin signaling in hepatocytes through BLT1 by activating the NFκB pathway and up-regulating inhibitors of insulin pathways such as Phosphatase and Tensin homolog (PTEN) and Protein-Tyrosine Phosphatase 1B (PTP1B) (31). Thus, LTB_4_ could promote insulin resistance by enhancing macrophage pro-inflammatory cytokine production, potentiating IL-1β action in insulin target organs and negatively affecting different components of insulin action. Therefore, LTB_4_ is an essential mediator in the development of insulin resistance in T2D.

Retinal capillary degeneration is a hallmark of diabetic retinopathy, and there is evidence that LTB_4_ is involved in this diabetes co-morbidity. This is supported by studies in animal models of diabetic retinopathy. 5-LO-deficient mice exhibited decreased leukocyte adherence to the vascular wall (the leukocyte subset was not assessed in this study), superoxide generation, NFκB expression and did not exhibit signs of capillary degeneration (32, 33). Both superoxide generation and NFκB expression can be induced by MyD88-dependent events (34). In humans, leukotriene precursor levels were increased in vitreous samples from patients with diabetic retinopathy compared with samples from non-diabetics (35). These results show that the 5-LO pathway is important for the development of diabetic retinopathy in humans.

In gout, joint deposition of monosodium urate (MU), a byproduct of purine degradation, is the disease etiological agent. MU is to activate macrophage NLRP3 leading to IL-1β and IL-18 secretion (caspase-1-dependent), IL-6, CXCL1 and CXCL2 production and inflammatory cell recruitment (36). It has been shown that LTB_4_ is produced by macrophages stimulated with MU and in the knee-joint of mice injected with MU crystals. Amaral et al. showed that pharmacologic and genetic inhibition of LTB_4_ production or BLT1 antagonism reduced MU-induced IL-1β and CXCL1 production and this correlated with neutrophil migration to the joint. Moreover, the injection of LTB_4_ into the joint was sufficient to induce IL-1β production and neutrophil recruitment, suggesting an essential role for this lipid mediator in the pathogenesis of gout (37). In patients, LTB_4_ in gouty effusion was found at a higher concentration that in synovial fluid from patients with rheumatoid arthritis or osteoarthritis (38).

In summary, involvement of LTB_4_ on sterile inflammation in metabolic diseases is supported by the finding that inhibition of LTs synthesis or BLT1 antagonism: (a) reduced IL-1β and TNF-α serum levels in T1D (15) and MCP-1, IL-6, and TNF-α serum levels in T2D (29); (b) reduced the sterile inflammation in adipose tissue in obese mice, more specifically the macrophage infiltration (28), pro-inflammatory cytokine production (10), and NFκB activation (10); reduced neutrophil migration and IL-1β production in a murine model of gout (37); prevented diet-induced insulin resistance and steatosis (28, 30), and reduced susceptibility to sepsis in T1D mice (15).

Evidence presented here led us to propose that LTB_4_ has a central role in metabolic dysfunctions. By increasing MyD88 expression, LTB_4_ enhances macrophage response to TLR/IL1 receptor agonists potentiating the sterile inflammation, a central event in metabolic disease progression. Furthermore, LTB_4_ can amplify tissue injury by increasing reactive oxygen and nitrogen species that are known to mediate β-cell destruction, impairing insulin production. Although further studies are required, inhibition of the LTB4/BLT1 axis is a promising therapeutic strategy for the treatment of metabolic disorders. There is a 5-LO inhibitor already approved to treat asthma, and BLT1 antagonists are under development. Reduction in LTB_4_ production or activity may reduce sterile inflammation and decrease disease severity.

## Conflict of Interest Statement

The authors declare that the research was conducted in the absence of any commercial or financial relationships that could be construed as a potential conflict of interest.

## Funding

Fundação de Amparo a Pesquisa do Estado de São Paulo (FAPESP) and the National Institute of Health (HL-103777-01 and HL-124159-01).
